# Applying Stretch to Evoke Hyperreflexia in Spasticity Testing: Velocity vs. Acceleration

**DOI:** 10.3389/fbioe.2020.591004

**Published:** 2021-02-16

**Authors:** Lizeth H. Sloot, Guido Weide, Marjolein M. van der Krogt, Kaat Desloovere, Jaap Harlaar, Annemieke I. Buizer, Lynn Bar-On

**Affiliations:** ^1^Amsterdam UMC, Vrije Universiteit Amsterdam, Department of Rehabilitation Medicine, Amsterdam Movement Sciences, Amsterdam, Netherlands; ^2^Institute of Computer Engineering (ZITI), Heidelberg University, Heidelberg, Germany; ^3^Department of Rehabilitation Sciences, KU Leuven, Leuven, Belgium; ^4^Department of Biomechanical Engineering, TU Delft, Delft, Netherlands; ^5^Emma Children's Hospital, Amsterdam UMC, Amsterdam, Netherlands

**Keywords:** spasticity assessment, stretch reflex, hyperreflexia, cerebral palsy, spastic paresis, upper motor neuron

## Abstract

In neurological diseases, muscles often become hyper-resistant to stretch due to hyperreflexia, an exaggerated stretch reflex response that is considered to primarily depend on the muscle's stretch velocity. However, there is still limited understanding of how different biomechanical triggers applied during clinical tests evoke these reflex responses. We examined the effect of imposing a rotation with increasing velocity vs. increasing acceleration on triceps surae muscle repsonse in children with spastic paresis (SP) and compared the responses to those measured in typically developing (TD) children. A motor-operated ankle manipulator was used to apply different bell-shaped movement profiles, with three levels of maximum velocity (70, 110, and 150°/s) and three levels of maximum acceleration (500, 750, and 1,000°/s^2^). For each profile and both groups, we evaluated the amount of evoked triceps surae muscle activation. In SP, we evaluated two additional characteristics: the intensity of the response (peak EMG burst) and the time from movement initiation to onset of the EMG burst. As expected, the amount of evoked muscle activation was larger in SP compared to TD (all muscles: *p* < 0.001) and only sensitive to biomechanical triggers in SP. Further investigation of the responses in SP showed that peak EMG bursts increased in profiles with higher peak velocity (lateral gastrocnemius: *p* = 0.04), which was emphasized by fair correlations with increased velocity at EMG burst onset (all muscles: *r* > 0.33–0.36, *p* ≤ 0.008), but showed no significant effect for acceleration. However, the EMG burst was evoked faster with higher peak acceleration (all muscles *p* < 0.001) whereas it was delayed in profiles with higher peak velocity (medial gastrocnemius and soleus: *p* < 0.006). We conclude that while exaggerated response intensity (peak EMG burst) seems linked to stretch velocity, higher accelerations seem to evoke faster responses (time to EMG burst onset) in triceps surae muscles in SP. Understanding and controlling for the distinct effects of different biological triggers, including velocity, acceleration but also length and force of the applied movement, will contribute to the development of more precise clinical measurement tools. This is especially important when aiming to understand the role of hyperreflexia during functional movements where the biomechanical inputs are multiple and changing.

## Introduction

Spastic paresis (SP) is the most commonly diagnosed impairment in children with cerebral palsy or hereditary spastic paraplegia, and the most common cause for physical disability in children (Cans et al., 2002). The goal of treatment of children with SP is to improve function, i.e., activities of daily life such as walking. SP is generally characterized by increased resistance to motion in affected joints, which can be caused by neural- and tissue related impairments (van den Noort et al., 2017). Neural impairments include exaggerated reflex responses and baseline muscle activation (Dietz and Sinkjaer, 2007; van den Noort et al., 2017). Tissue-related impairments often develop over time, and include muscle shortening and increased stiffness of muscle fiber, tendon, or connective tissue (Dietz and Sinkjaer, 2007). Even though the underlying etiology guides treatment selection to manage SP, the ability to accurately measure these impairments and especially the exaggerated reflex responses, remains challenging.

The commonly accepted definition of spasticity refers to a velocity-dependent increase in tonic stretch reflexes (muscle tone) resulting from hyper-excitability of the stretch reflex (Lance, 1980), or hyperreflexia. Following this definition, clinical scales such as the Modified Tardieu Scale provide a qualitative assessment of spasticity by moving a passive joint at low and high velocity while grading the resistance to the movement (Gracies et al., 2010). The resistance during slow movement is suggested to be indicative of increased tissue stiffness and/or baseline muscle activation, while the difference in resistance between slow and fast movement is thought to reflect hyperreflexia. To standardize this procedure and increase the objectivity of the spasticity assessment, instrumented manual and robotic versions have been proposed (Wood et al., 2005; Bar-On et al., 2014). These instrumented tests provide not only quantitative information on evoked muscle activation, but also control or can provide feedback on the applied movement profile to stretch the muscle, yielding better accuracy and reliability than manual subjective scales (Burridge et al., 2005).

Robotic devices are increasingly applied to evoke and record muscle responses while controlling either the applied velocity or the applied torque (Pierce et al., 2006; Poon and Hui-Chan, 2009; de Gooijer-van de Groep et al., 2013; Willerslev-Olsen et al., 2013; Sloot et al., 2015). These standardized tests are an important step toward quantification of evoked abnormal muscle activation, although most setups still operate under the assumption that hyperreflexia is solely dependent on a velocity-driven feedback loop. Different mechanisms however are known to regulate reflex responses: the muscle spindles, which are sensory proprioceptors in the skeletal muscles that sense muscle stretch and rate of change, and Golgi tendon organs that sense force or tension. Despite a multitude of research on hyperreflexia, there is still no consensus on the exact feedback mechanisms responsible for the stretch reflexes measured during clinical tests. One reason is the wide variability in how the motorized movement trajectories are applied even for specifically the ankle joint (Wood et al., 2005). For example, stretches are applied at different starting points or range of ankle angles, while the sensitivity to activate a stretch reflex is found to be dependent on the muscles' starting length (Meinders et al., 1996). Research on hyperreflexia does suggest two main pathophysiological characteristics of recorded muscle activity during the spasticity assessments: a reduced excitability threshold and exaggerated response intensity (Sheean, 2008). The effects of different biomechanical triggers, such as position, velocity, and acceleration of the applied stretch on these characteristics of the evoked muscle response are still unknown.

As motor-controlled devices allow for controlled replication of the movement profiles applied during the clinical examination, they provide a unique testbed to disentangle the effect of different biomechanical triggers on abnormal muscle responses and are thus crucial for correct interpretation of the clinical tests (Wood et al., 2005). Previous research found different amounts of ankle plantar flexor muscle activation during instrumented motor-controlled assessment of hyperreflexia compared to manual techniques (Rabita et al., 2005; Sloot et al., 2017). As dorsiflexion angle and peak velocity did not differ between methods, other differences in movement trajectories were suggested to have affected the muscle's responses. Specifically, bell-shaped velocity profiles were applied during the manual assessments while the robotic assessments applied ramp-and hold movements with high accelerations. Since bell-shaped velocity profiles are found to be more representative of functional movement such as walking, it is relevant to explore the role of velocity vs. acceleration components in evoked muscle responses during standardized motorized assessment.

Thus, it is still speculative what mechanisms are triggered during clinical examination and the role of different movement profiles on evoking abnormal muscle activation. Therefore, the aim of this study was to compare different bell-shaped movement profiles, currently most resembling functional movement, to examine the effect of increasing imposed rotational velocity vs. acceleration on the evoked calf muscle responses. To verify the abnormal characteristics of the muscle response measured in children with SP it was contrasted to the response in typically developing children. This work contributes to the understanding of the influence of the applied movement profile during manual clinical spasticity examination and as such, supports the development of clinically relevant instrumented alternatives.

## Methods

A convenience sample of 13 children with SP [7 female; 11.3 ± 8.2 yr; 32.4 ± 10.0 kg; gross motor function classification system (GMFCS, Palisano et al., 1997) level I (*n* = 3), II (*n* = 9), and III (*n* = 1); spasticity test (SPAT, Scholtes et al., 2007) score, executed with extended knee, of 0 (*n* = 3), 1 (*n* = 4), and 2 (*n* = 6)] and 8 typically developing children (5 female; 9.9 ± 1.6 yr; 29.6 ± 19.0 kg) were included in the study. Potential participants were excluded when reporting medical problems or sports injuries interfering with lower leg joint mechanics in either group and in the SP group if treatment occurred in the previous 6 months with Botulinum NeuroToxin-A, any neuro- or orthopedic surgery involving the lower-leg, a baclofen pump, if there was more than 20° knee flexion contracture or severe cognitive deficits. Informed consent was provided by the legal guardians and ascent obtained from the participants. The study was approved by the Dutch central ethical committee on research involving human subjects (CCMO) [NL4441073.000.12].

### Protocol

**Figure 1:**
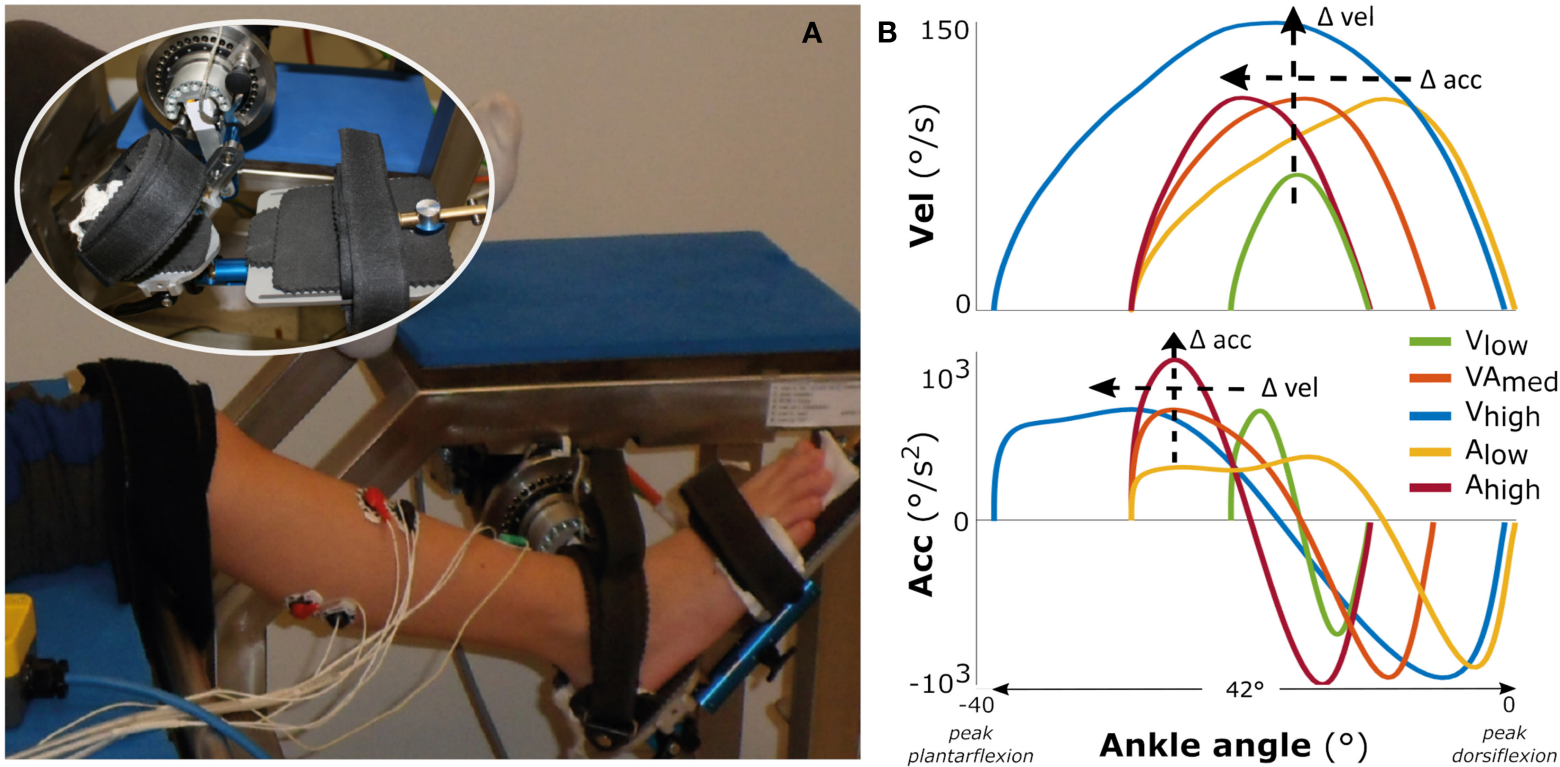
Measurement set-up and movement profiles. On the left **(A)** the motorized footplate with adjustable chair, with the foot fixation enlarged as inset. On the right **(B)** the imposed movement velocity (upper figure) and the imposed movement acceleration (lower figure) vs. the ankle range of motion, averaged over all participants.

Children were seated in an adjustable chair with the hip and knee joints set to 120° hip flexion and 20° knee flexion for the most affected leg in the SP group (as defined by records of their clinical examination) and the right leg in the TD group (Figure 1A). The foot was optimally fixated using an adjustable footplate that allowed stabilization of the subtalar joints (Huijing et al., 2013). The footplate was attached to a motor that applied rotations around the talo-crural joint in the sagittal plane. The rotation was measured using a potentiometer and the position corresponding to the individual's neutral ankle angle was calibrated using a goniometer (MOOG, Nieuw-Vennep, The Netherlands) (Sloot et al., 2015). The axis of the talo-crural joint and the motorized footplate were visually aligned by minimizing knee translation during manual rotation of the ankle in the footplate. Ankle passive range of motion (ROM) was determined by imposing low velocity dorsal (max. 6–10 Nm) and plantar (max. 4–7.5 Nm) flexion moments as measured by an integrated force transducer (Sloot et al., 2015). Surface EMG electrodes were placed on the gastrocnemius medialis (GM), lateralis (GL), soleus (SO), and tibialis anterior (TA) muscles according to SENIAM guidelines (Hermens et al., 2000) and measured with a wired system (Porti7, TMSi, Oldenzaal, The Netherlands).

The experimental measurements consisted of two repetitions of five different bell-shaped movement profiles imposed over 42° of ankle range of motion (Figure 1B), lasting between 0.46 and 0.73 s. The profiles had three levels of maximum acceleration (low: 500°/s^2^; medium: 750°/s^2^; high: 1,000°/s^2^) and three levels of maximum velocity (low: 70°/s; medium: 110°/s; high: 150°/s) (see Supplementary Table 1). For comparison between movement profiles, the starting ankle plantarflexion orientation was adjusted such that each profile was carried out within the same 42° range (this range was required to reach the highest velocity), to ensure that the peak velocity or acceleration were reached at similar joint positions between profiles (Figure 1B). This range ended at the subject-specific maximal ankle dorsiflexion angle, which corresponded to mean ± std: 7.0 ± 16.0° TD vs. −4.6 ± 8.7° SP (Figure 1B; see Supplementary Figure 1 for the variability in absolute ankle angle range between participants and more details about duration of the profiles). With this approach, the movement profiles were always applied over a range that ensured muscle stretch, in contrast to applying the profiles at a certain, non-individualized ankle angle. Measurements started at a random time instant, with at least 20 s rest in between imposed stretches to account for possible time-dependent viscosity effects and to allow participants to return to a relaxed state. The children were instructed to remain relaxed, and a stretch was repeated if any EMG activation of agonist and/or antagonist occurred before or at an unexpected time during stretch.

### Analysis

Sagittal ankle angle, foot reaction moment, and muscle activity were measured at 1,024 Hz. EMG data were filtered using a band pass filter (20–500 Hz), a notch filter (45–54 Hz) and a low-pass filter (100 Hz), after which the root-mean-square envelope was extracted. Ankle angle and moment were low-pass filtered at 30 Hz. All filters were 6th order bi-directional Butterworth implementations.

In line with other instrumented tests that mimic clinical examinations (Bar-On et al., 2014; Sloot et al., 2017), the amount of muscle activation was quantified by both the average and peak EMG measured during the imposed movement profile, after subtraction of the baseline (i.e., minimum EMG measured 0.5 s prior to start of the stretch). The peak was taken as the 95th percentile to correct for outliers.

In addition to this general evaluation of the amount of muscle activation, we explored the characteristics of potentially evoked hyperreflexia in SP specifically. First, muscle responses were detected for each triceps surae muscle according to the method of Staude and Wolf (1999) that identifies bursts in the EMG signal, with the additional condition of peak EMG exceeding two standard deviations of the signal (Sloot et al., 2017). The identified EMG bursts were visually inspected and manually corrected if needed. Only trials with the occurrence of an EMG burst were further analyzed. For each detected EMG burst, we evaluated the time until EMG burst onset as well as the intensity of the response. The absolute time to onset was defined from the moment at which the applied movement was initiated until onset of the burst. It should be noted that this is not quantifying the latency of the evoked muscle response, as the timing of the actual threshold that triggers a reflex response is unknown. The intensity of the response was defined as the burst peak EMG, i.e., the maximum EMG after subtraction of the baseline value, recorded in the time window between EMG burst onset plus 50 ms, a sufficient time window to capture stretch reflex activation (Willerslev-Olsen et al., 2014), and normalized to each muscle's peak value found over all profiles to allow for comparison between individuals. To substantiate this analysis, we also identified the velocity and acceleration at the onset of the EMG burst—minus 30 ms, to account for an electromechanical delay (Sinkjaer et al., 1999).

### Statistics

To evaluate the effect of velocity and acceleration on the amount of muscle activation, average and peak EMG parameters were averaged over the two repetitions per profile. As these variables did not follow a normal distribution, non-parametric Friedman tests were performed per population (TD and SP) to compare between the velocity profiles (VEL: low, medium, high) and similarly between the acceleration profiles (ACC: low, medium, high). *Post-hoc* testing was performed using Tukey-Kramer multiple comparison correction. To test between TD and SP, data was concatenated over profiles per variable and compared using the non-parametric Wilcoxon sign-rank test.

For the detailed analysis of EMG bursts in the SP group, the effects of acceleration and velocity profiles on both time until onset of the EMG burst and on peak EMG burst were assessed as previously, including *post-hoc* testing. For the assessment of the velocity effect, onsets that occurred after the applied peak velocity plus the electromechanical delay (<4% of the cases) were not considered. Similarly, for the acceleration effect, onsets that occurred after applied peak acceleration plus electromechanical delay (<30% of the cases) were not considered. We confirmed that these exclusions did not affect the conclusions of this analysis. To substantiate the profile analysis for peak EMG bursts, we also correlated the individual peak EMG burst values to both the corresponding velocity and acceleration at the onset of the EMG burst using Spearman's correlation analysis. Correlation values >0.6 were considered good; 0.41–0.60 moderate; 0.21–0.40 fair; and <0.20 poor (Altman, 1991). Level of significance was set at *p* = 0.05. All analyses were performed in Matlab (R2019a, Natick, MA, USA).

## Results

### Amount of Muscle Activity

**Figure 2:**
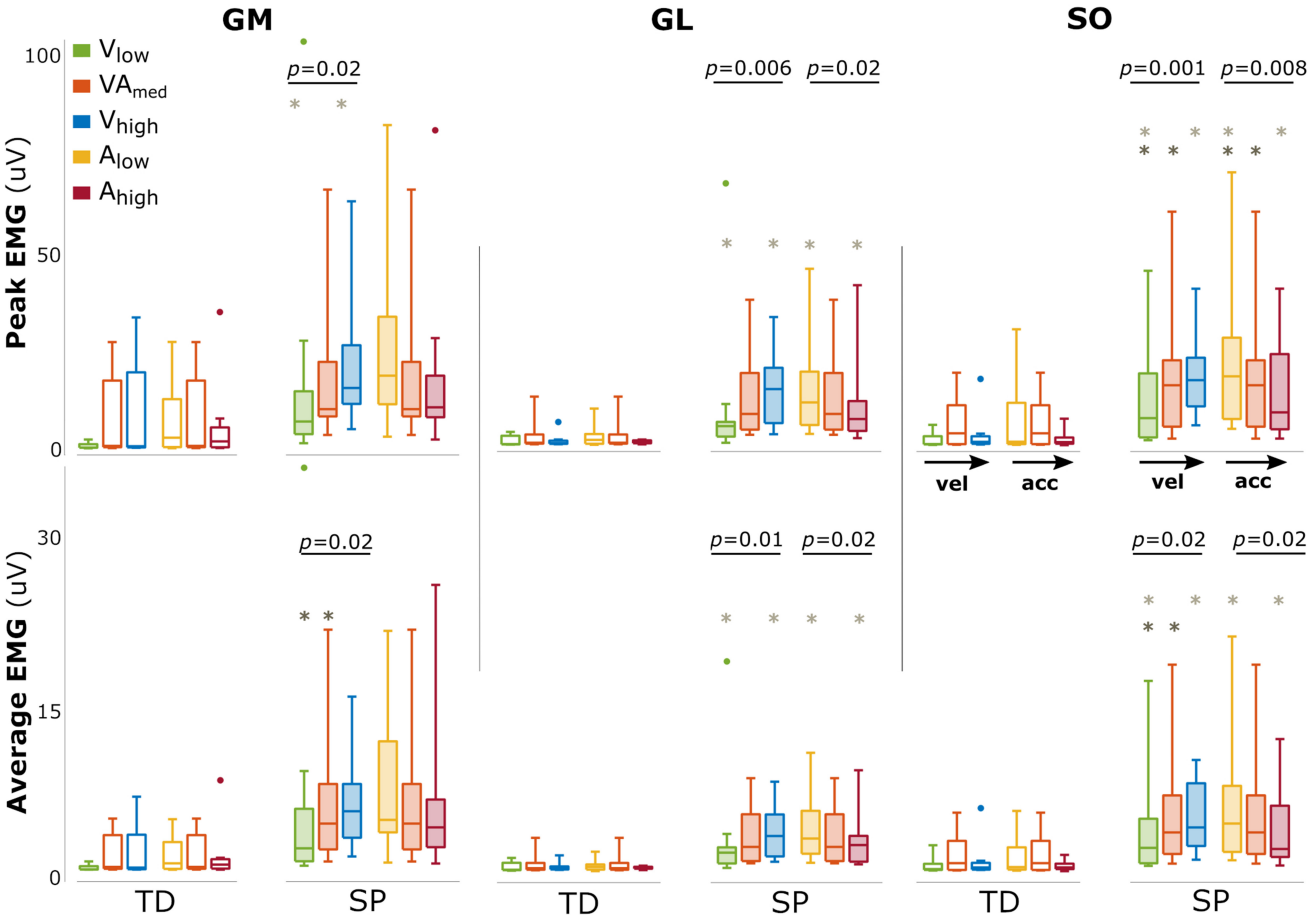
Changes in amount of muscle activation with changing movement velocity and acceleration. Bars are shown for typically developing children (TD, open bars) and children with spastic paresis (SP, filled bars) for increasing movement velocity (green, orange, blue) and acceleration (yellow, orange, red). The central mark indicates the median in each bar plot, the bottom and top edges of the box the 25th and 75th percentile, the whiskers extend to the most extreme data points not considered outliers, which are indicated with small dots. *P*-values of group effects and *post-hoc* results are indicated (dark gray asterisk: difference between low and medium; light gray asterisks: between low and high).

The amount of evoked muscle activation was significantly higher for the children with SP compared with TD children in all triceps surae muscles (all *p* < 0.001), underlining the exaggerated response in SP (Figure 2). In typically developing children, we did not find an effect of either velocity or acceleration on the amount of muscle activation (all *p* > 0.19).

In SP, larger amounts of muscle activation were found between movement profiles with increasing movement velocity (both peak and average for all muscles: *p* < 0.02, Figure 2). The opposite effect was found for acceleration, with a reduced amount of triceps surae muscle activation between movement profiles with increasing movement acceleration, most prominently for GL and SO (GL_peak_: *p* = 0.02; GL_mean_: *p* = 0.02 SO_peak_: *p* = 0.008; SO_peak_: *p* = 0.02, Figure 2). An overview of these parameters and statistical results can be found in Supplementary Table 2.

### SP: EMG Bursts

**Figure 3:**
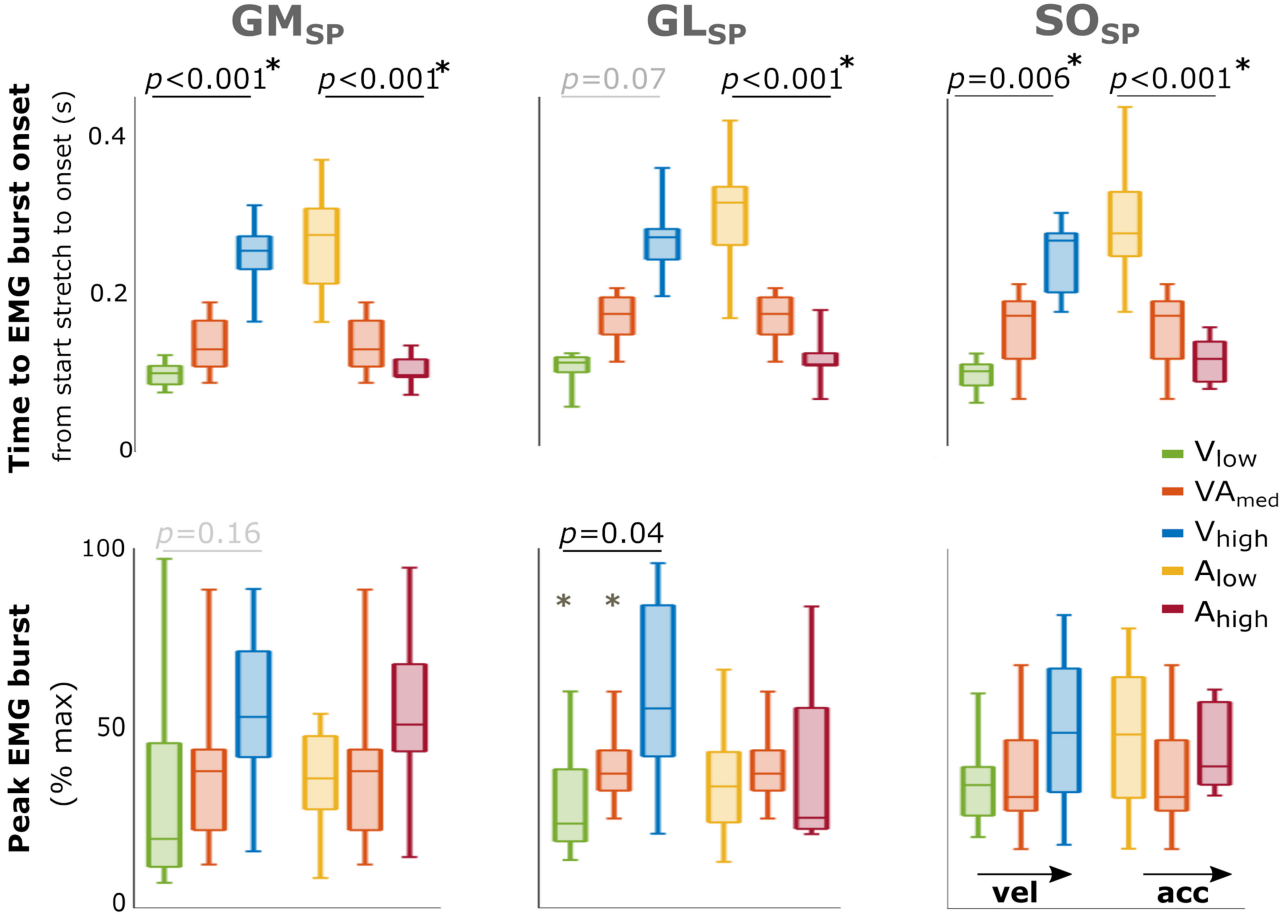
Effect of velocity and acceleration on EMG burst characteristics in SP. Effect of increasing velocity (green, orange, blue) and acceleration (yellow, orange, red) on both total time from start stretch initiation to EMG burst onset (top) and peak EMG burst (bottom). The central mark indicates the median in each bar plot, the bottom and top edges of the box the 25th and 75th percentile, the whiskers extend to the most extreme data points not considered outliers, which are indicated with small dots. *P*-values of group effects and *post-hoc* results are indicated (star next to *p*-value indicates all three conditions are significantly different). With GM gastrocnemius medialis, GL gastrocnemius lateralis and SO soleus muscle.

EMG bursts were detected in 73–92% of the trials for the three triceps surae muscles in SP, and the percentages were comparable between movement profiles (Supplementary Table 3). Comparisons between different movement profiles indicated that peak EMG burst increased with increasing peak velocity for GL (*p* = 0.04) with a similar trend for GM (*p* = 0.16) but showed no significant differences between acceleration profiles (Figure 3). The time until EMG burst onset increased between movement profiles with increasing velocity (GM: *p* < 0.001; SO: *p* = 0.006; a trend for GL: *p* = 0.07; Figure 3) but decreased between movement profiles with increasing acceleration (all muscles *p* < 0.001). An overview of these parameters and statistical results can be found in Supplementary Table 4.

**Figure 4:**
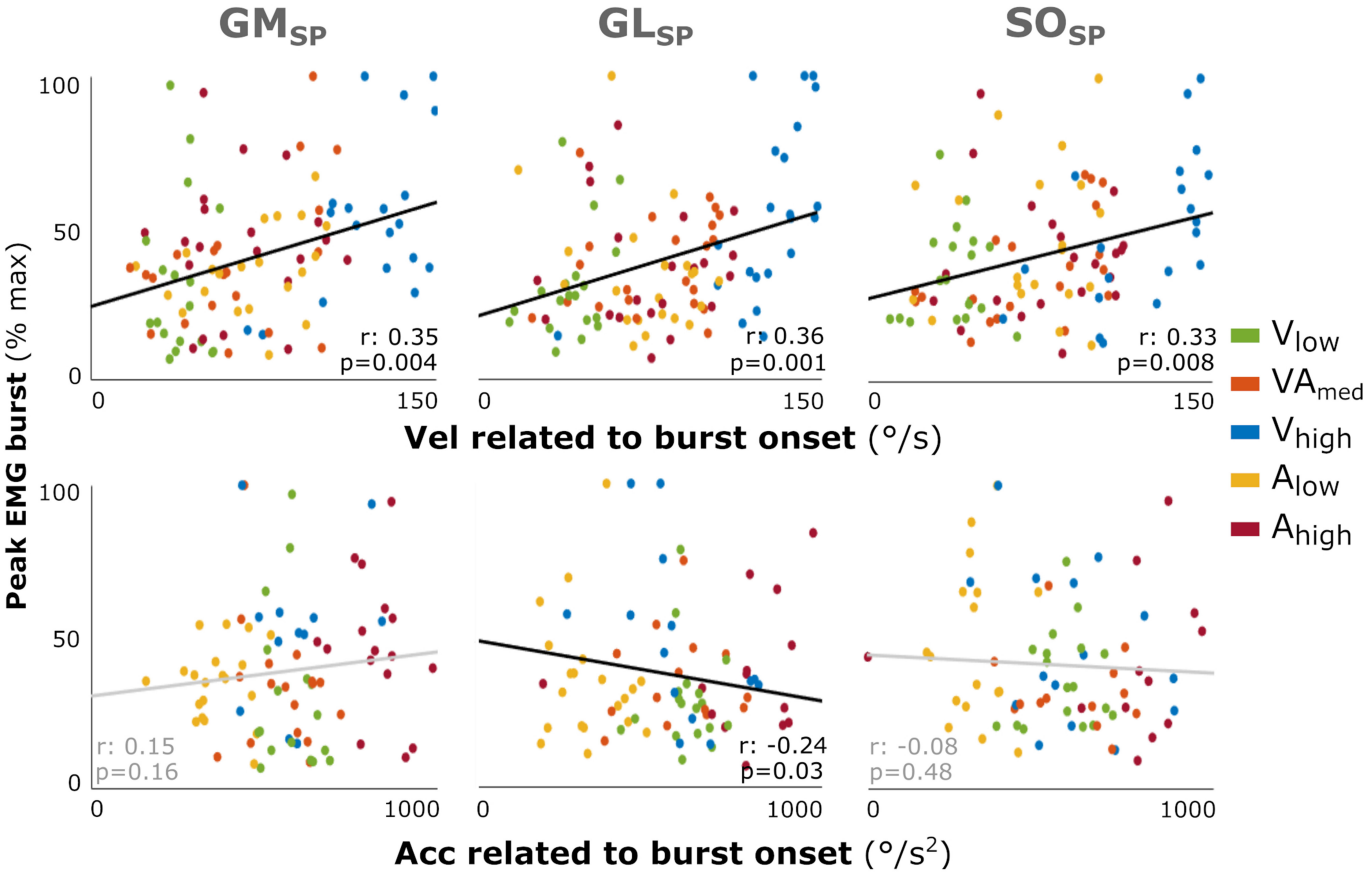
Correlation between the muscle response intensity and velocity or acceleration related to burst onset in SP. Individual intensity peak EMG values are correlated with the acceleration (Acc) or velocity (Vel) values at the time of EMG burst onset minus the electromechanical delay (30 ms). Peak burst EMG values are the maximum EMG minus the baseline, normalized to each muscle's peak over all profiles per participant. Although not affecting the analysis, the color coding for the different movement profiles is used for context. Spearman's rho correlation coefficients and corresponding *p*-values are given. With GM gastrocnemius medialis, GL gastrocnemius lateralis, and SO soleus muscle.

As individual variability combined with reduced power in this EMG burst analysis might have masked a relation between peak EMG burst and applied velocity and acceleration in the profile analysis, as found for the total amount of muscle activation, we performed an additional correlation analysis using individual data points. Increased peak EMG burst was fairly correlated with higher velocity at onset of the EMG burst for all three muscles (GM: *r* = 0.35; GL: *r* = 0.36; SO: *r* = 0.33; all: *p* < 0.008; Figure 4). In contrast, there was none or a negative correlation with acceleration at onset of the EMG burst (GL: *r* = −0.24, *p* = 0.003; GM: *r* = 0.15; SO: *r* = −0.08).

## Discussion

Current clinical spasticity assessments impose movements on relaxed muscles to evoke exaggerated stretch reflexes that resist the imposed muscle stretch. Instrumented versions of these tests improve the quantification of the outcome. Yet, it remains the question whether the complex physiological mechanisms underlying the exaggerated reflex responses to stretch are fully understood and thus correctly assessed. As expected, we found that the amount of ankle plantar flexor muscle activation was larger and sensitive to the profiles in SP compared with TD, with the amount increasing with increasing peak velocity but decreasing with increasing peak acceleration. A more detailed analysis of activation bursts in SP revealed that the intensity of the response (peak EMG burst) increased between movement profiles with increasing maximum velocity. This was also confirmed by the correlation analysis of individual data points whereby higher threshold velocities correlated with more intense responses. On the other hand, EMG bursts were evoked faster with higher peak acceleration.

The increase in the amount of muscle activation with increasing stretch velocity in SP suggests the presence of an underlying velocity-dependent increase in tonic stretch reflexes according to the definition of spasticity by Lance (1980). The velocity dependence of stretch reflexes is originally based on findings from animal studies, showing increased velocity-sensitive fusimotor drive at the muscle spindles' gamma motor fibers in spastic muscles. However, fusimotor drive does not seem to be increased in human spastic muscles (Sheean, 2008), and unlike our findings, velocity-related reflex activation in spastic muscles has also been shown to be weak and non-linear (Powers et al., 1989; Blum et al., 2017; Baude et al., 2019). Reduced or dysregulated inhibitory drive to the alpha motor neurons from the spine and brain has also been suggested to underlie spasticity, although the precise relation to changed excitability and sensitivity of the stretch reflex remains unknown. Thus, the actual origin of the generally accepted velocity-dependency in spasticity is still unclear.

In our more detailed analysis of EMG bursts in SP we specifically investigated only those trials in which there was a detectable burst in the EMG, commonly regarded as indicative of hyperreflexia. Such EMG bursts were detected in most SP participants and the number of bursts did not seem to be profile- or muscle-dependent. We explored two characteristics of the recorded muscle activity: the time to EMG burst onset (not equivalent to reflex latency), and the intensity of the response represented by the peak EMG burst. In the context of hyperreflexia, the timing may represent hyper-excitability or the susceptibility of the muscle to motor neuron recruitment, while response intensity may represent hypersensitivity, or the number of recruited motor neurons (Bar-On et al., 2015). We found earlier EMG burst onsets in profiles with higher accelerations, while profiles with higher velocities delayed the onsets. On the other hand, we found that the intensity of the response increased in profiles with higher velocity without an effect of acceleration. This was substantiated by the fair correlations indicating more intense responses with higher threshold velocities. These findings suggest that if we indeed measured stretch reflex responses, hyper-sensitivity (peak EMG burst) is velocity-dependent, while hyper-excitability (time to EMG burst onset) of the muscle response is driven by acceleration.

Evidence for acceleration-driven stretch reflex activation has been previously reported in studies investigating triceps surae activity during postural responses. In particular, the onset of EMG bursts following muscle stretch due to standing perturbations have repeatedly been found to be related to acceleration (Schafer, 1967; Finley et al., 2013; Blum et al., 2017). It is interesting to note that while the applied acceleration did affect our reported total amount of evoked muscle activity, which is often analyzed in instrumented spasticity tests, this biomechanical trigger is usually not accounted for. Our reported relation between onset of burst EMG and acceleration also corresponds to a recent study in SP showing that an acceleration-based model accurately predicted the timing of GM EMG burst onset (Falisse et al., 2018). However, these authors also showed that a force-related model accurately predicted both EMG burst onset timing as well as the response intensity. Advocates of the force-feedback mechanism argue that muscle spindle sensitivity depends on the state of the cross-bridges prior to muscle stretch. A greater cross-bridge overlap will increase a muscle's initial tension, affecting the detection and thus response to the proceeding stretch (Blum et al., 2017). Since the velocity-profiles in our study had small differences in starting position (max. 19°), it is possible that this resulted in small variations in pre-tension and thus in muscle activation (De Groote et al., 2018). However, these history-dependent effects on stretch reflex activation are probably more relevant when muscles are active, as the amount of cross-bridge overlap depends on the performed activity. Falisse et al. took a first step at understanding reflexes during walking, showing that modeled force-dependency was also a better predictor of exaggerated gastrocnemius muscle activity recorded during early stance in children with SP (Falisse et al., 2018). Therefore, acceleration and force-related variables may be important to evaluate in addition to velocity when developing accurate instrumented spasticity tests, especially when assessing functional movements.

While we showed relations between the evoked muscle responses and both stretch velocity and acceleration, it is important to note that stretch reflexes are also known to be length-dependent. Wu et al. showed that at faster velocities, the joint was moved further in its position to a longer muscle length at catch (Wu et al., 2010). In the current study, profiles with higher velocities were started more toward plantarflexion such that maximum velocity occurred at a similar joint position (Figure 1B). Therefore, the longer time to EMG burst onset with increasing velocity may have been related to it taking longer for the muscle to be sufficiently stretched. In addition, in order to apply each profile over the same angular range, profiles differed in the maximum reached dorsiflexion angle. The profiles of A_low_ and V_high_ both ended at a similar maximum angle and showed similar peak EMG burst responses. Although to a lesser extent, this was also the case for A_high_ and V_low_ profiles. These findings may be indicative of some length-dependency. Meinders et al. argue that at longer lengths, the GM stretch reflexes are reduced (Meinders et al., 1996), possible due to inhibitory actions following stimulation of the Golgi tendon organ. It should be noted that standardizing the joint angle might not precisely standardize the input at the level of the muscle spindles, as the effects of ankle joint displacement in particular in the pennate triceps surea muscles have been shown to not linearly translate to muscle-tendon complex and thus muscle fiber lengthening (Weide et al., 2020). Therefore, we chose to align the peak applied velocities and accelerations at about the same ankle angle and apply the movement profiles over an ankle range at which an individual's muscles were (sufficiently) lengthened. Nevertheless, given the relevance of length-dependency on stretch reflexes, future investigations applying bell-shaped movement profiles should also vary for muscle length and examine the role of start and end muscle length in current manual clinical spasticity tests, for instance by using a measured passive angle-force relationship during a slow profile to indicate the start of muscle stretch.

The nature of both manual clinical and instrumented tests assumes that the evoked muscle activity reflects involuntary reflex activation rather than voluntary muscle contraction. This requires that the participant actively relaxes, which is a somewhat artificial situation. Also, since longer lasting stretches are applied over a large ROM during these tests, other feedback mechanisms than stretch reflexes, such as via the supraspinal structures, and fast voluntary responses might occur. As we did not apply instantaneous perturbations, the exact biomechanical trigger and thus latency of a possible (reflex) response are unknown. The lack of this information makes it challenging to determine the exact nature of the measured muscle response, given that the shortest voluntary reaction time of 100 ms and supraspinal structure reaction time of 70–80 ms fall within the measured time window (Sinkjaer et al., 1988; Mirbagheri et al., 2000). However, we do have several reasons to assume we are analyzing involuntary muscle responses. First, the children were instructed to relax. Younger children, who had problems actively relaxing their muscles, were enticed to do so by playing a “statue-still” game, also involving their parents. We did monitor the background EMG before and during applied stretch and repeated trials with higher levels of activity prior to stretching. Unfortunately, no study has yet reported an approach to verify or quantify the state of muscle relaxation or the occurrence of voluntary reactions during these types of clinical tests, other than subjective impressions and EMG monitoring. Second, conform the presence of abnormal activation, bursts in muscle responses were more common in SP compared to TD. Voluntary anticipatory responses are expected to result in the opposite effect because lowered sensory drive that is required to produce these movements, has been found in patients with neurological lesions (Nielsen et al., 2020). Some studies have applied very short (up to 50 ms) perturbations to evoke muscle responses, which allows for more accurate determination of the underlying neurophysiological mechanisms of stretch reflexes and the latencies of different peaks (Sinkjaer et al., 1988; Mirbagheri et al., 2000). Such an approach could provide more insight into the relation between the different triggers examined in this study and monosynaptic Ia stretch reflexes (usually referred to as M1, with an onset around 30 ms), indirect spinal pathways (M2, around 60 ms), transcortical reflexes (M3, around 90 ms) or later responses that are not purely reflex in nature. However, as these perturbations are applied at a specific ankle angle or muscle length, their relation to current clinical tests performed over the whole ROM, or to functional movements, are unclear. Understanding the interplay between different biomechanical triggers and the mechanisms underlying hyperreflexia as well as voluntary contractions warrants more emphasis, especially in the light of the development of instrumented tests.

Next to the limitations arising from the nature of functional tests, the current study has some specific limitations. First, we applied lower maximum velocity and acceleration than the values up to 300 and 3,600 deg/s^2^ previously reported in literature for manual stretching methods (Berardelli et al., 1983). In our study maximum values were chosen to ensure the children were able to remain relaxed during the measurements and were found to be high enough to evoke stretch reflexes in most of the children with SP. Second, only three different levels of velocity and acceleration were included in the study. While more intermediate steps would have increased the levels of detail, it is not expected to have changed the reported relationships. Third, we did not execute the different movement profiles in random order. However, having found opposite results for the effects of velocity and acceleration on stretch reflex parameters, while both were applied in increasing order, showed that the effect of the biomechanical triggers overruled any potential order effect. Third, the number of subjects included in this clinical study was limited, though large enough to find effects. Having included subjects with varying degrees of clinically diagnosed spasticity, we demonstrated that our findings can be cautiously generalized to a heterogeneous SP population. Our choice to also include mildly involved children with SP meant that not every profile had an EMG burst. Thus, the secondary analyses were carried out on fewer data, which may explain the findings of only fair correlations and may indicate that the reported effects may have even been underestimated. Finally, to ensure the analyses remained hypothesis driven, we excluded those instances when EMG burst onset occurred after peak velocity or acceleration similar as done in other studies (Falisse et al., 2018), but we confirmed that this did not affect the conclusions.

This study is the first to vary and control both the applied velocity and acceleration during an instrumented spasticity assessment. As expected, triceps surae activation was found to be larger and more sensitive to changes in velocity and acceleration in children with SP compared to TD children. In addition, the analysis in SP suggests that the two characteristics of hyperreflexia are affected by different biomechanical triggers: exaggerated response intensity seems linked to stretch velocity while higher acceleration seems to evoke faster responses. As such, this work indicates that biomechanical triggers should be accounted for when developing instruments to quantify spasticity. This becomes even more paramount when studying stretch reflex responses during functional movements in which both velocity and acceleration inputs are highly volatile. The reported differences in evoked muscle response between movement profiles indicate that any feedback provided by a manual instrumented test on applied stretch velocity should ultimately be accompanied by information on characteristics such as the acceleration. As this feedback will be complex and all the biological triggers are difficult to control, algorithms that extract and differentiate the response based on a range of stretches, or motorized alternatives to standardize the applied stretch, might be desirable. The testbed used in this study could be further refined to better identify the nature of the abnormal responses as well as control other factors affecting the stretch reflex, such as muscle length and force characteristics.

## Data Availability Statement

The raw data supporting the conclusions of this article will be made available by the authors, without undue reservation.

## Ethics Statement

The studies involving human participants were reviewed and approved by Dutch central ethical committee on research involving human subjects (CCMO) [NL4441073.000.12]. Written informed consent to participate in this study was provided by the participant's legal guardian/next of kin and ascent given by the participants.

## Author Contributions

LS, GW, KD, JH, MK, and LB conceptualized the methods. LS, GW, and LB designed the experiments. JH, AB, and LB acquired funding. GW and LB collected the data. LS and LB processed and analyzed the data. LS, MK, AB, and LB interpreted the data. LS and LB wrote the manuscript. LS generated the figures and tables. All authors provided critical feedback on the manuscript.

## Conflict of Interest

The authors declare that the research was conducted in the absence of any commercial or financial relationships that could be construed as a potential conflict of interest.

## Supplementary Material

Supplementary Figure 1 Imposed movement velocity profiles. **(A)** Variability between participants in the range of ankle angle over which the different movement profiles were applied. The average applied velocity vs. angle is shown for all movement profiles per group, with SP in solid lines and TD in dashed lines. The movement profile of V_high is also shown for each participant, representing the maximum angular range over which the profiles were applied. The zero angle represents the neutral ankle angle, with negative values being plantarflexion and positive values dorsiflexion. **(B)** The average movement profiles of the CP group shown over time, with the total duration of the profiles and timing of peak velocities.

Supplementary Table 1 Peak velocity and acceleration imposed during different movement profiles for typically developing (TD) children and children with spastic paresis (SP). A slow profile was used to determine the ankle range of motion of participants. With Max. maximum, Med. Medium, vel velocity, acc acceleration.

Supplementary Table 2 Effects on the amount of muscle activation for typically developing (TD) children and children with spastic paresis (SP). Median and interquartile range values per group as well as statistical outcomes. *Post hoc* results are indicated when p<0.05 (with Tukey-Kramer correction for multiple comparison), with lm representing a significant difference between the low and medium conditions and lh a difference between the low and high conditions (no differences were found for mh). Results for testing between the grouped data of TD and SP are also given. With GM, Gastrocnemius Medialis; GL, Gastrocnemius Lateralis; SO, soleus muscles.

Supplementary Table 3 Percentage of detected EMG onsets in children with spastic paresis (SP). Percentage of onsets taken over all trials in the SP data per profile. With GM, gastrocnemius medialis; GL, gastrocnemius lateralis; SO, soleus muscle.

Supplementary Table 4 Effects on EMG burst characteristics in children with spastic paresis. Median and interquartile range values for group effects on acceleration and velocity as well as statistical outcomes. *Post hoc* results are indicated when p<0.05 (with Tukey-Kramer correction for multiple comparison), with lm representing a significant difference between the low and medium conditions, lh a difference between the low and high conditions and mh between medium and high. With max. GM, Gastrocnemius Medialis; GL, Gastrocnemius Lateralis; SO, soleus muscles.
