# Supplementary figures and images for: Applying Stretch to Evoke Hyperreflexia in Spasticity Testing: Velocity vs. Acceleration
Source: Front Bioeng Biotechnol. 2021 Feb 16;8:591004. doi: 10.3389/fbioe.2020.591004 (PMC7921693; doi:10.3389/fbioe.2020.591004)

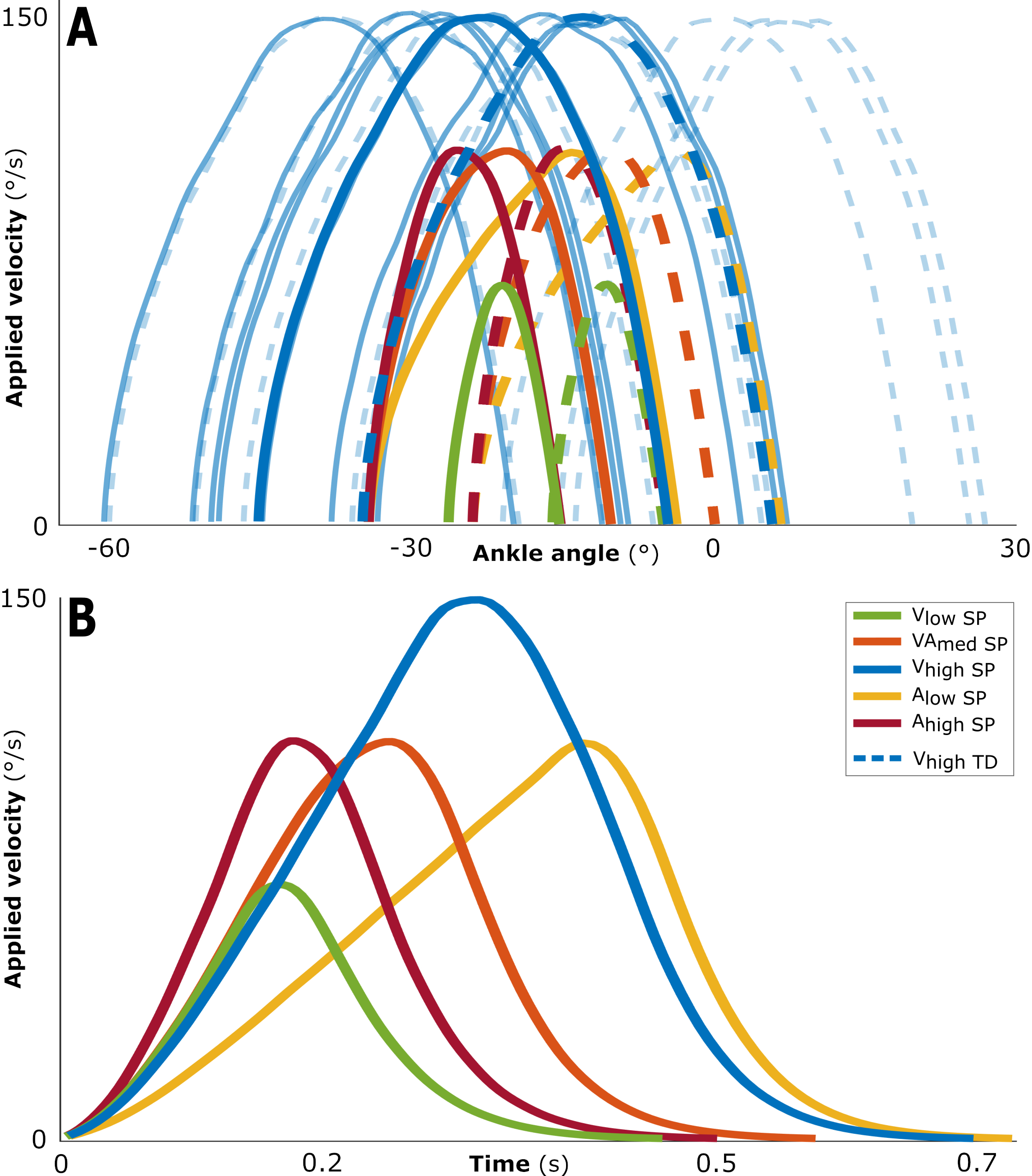

Supplement: Supplementary Figure 1 — Imposed movement velocity profiles. (A) Variability between participants in the range of ankle angle over which the different movement profiles were applied. The average applied velocity vs. angle is shown for all movement profiles per group, with SP in solid lines and TD in dashed lines. The movement profile of V_high is also shown for each participant, representing the maximum angular range over which the profiles were applied. The zero angle represents the neutral ankle angle, with negative values being plantarflexion and positive values dorsiflexion. (B) The average movement profiles of the CP group shown over time, with the total duration of the profiles and timing of peak velocities. [file Image_1.JPEG]
