# Supplementary material for: Applying Stretch to Evoke Hyperreflexia in Spasticity Testing: Velocity vs. Acceleration
Source: Front Bioeng Biotechnol. 2021 Feb 16;8:591004. doi: 10.3389/fbioe.2020.591004 (PMC7921693; doi:10.3389/fbioe.2020.591004)
Supplement: Supplementary Table 1 — Peak velocity and acceleration imposed during different movement profiles for typically developing (TD) children and children with spastic paresis (SP). A slow profile was used to determine the ankle range of motion of participants. With Max. maximum, Med. Medium, vel velocity, acc acceleration. [file Table_1.docx]

Supplementary Material

**Table 1: Peak velocity and acceleration imposed during different movement profiles for typically developing (TD) children and children with spastic paresis (SP).**

|  | | **SLOW** | | **ACC_LOW** | | **A/V_MED** | | **ACC_HIGH** | | **VEL_LOW** | | **VEL_HIGH** | |
| --- | --- | --- | --- | --- | --- | --- | --- | --- | --- | --- | --- | --- | --- |
| **Parameter** | **Unit** | Mean | [std] | Mean | [std] | Mean | [std] | Mean | [std] | Mean | [std] | Mean | [std] |
| **TD** | | | | | | | | | | | | | |
| Max. vel | [°/s] | 10.2 | [0.0] | 110.3 | [0.4] | 110.8 | [0.4] | 111.3 | [0.6] | 71.1 | [0.3] | 150.9 | [0.7] |
| Max. acc | [°/s^2^] | 24.5 | [0.9] | 491.4 | [8.0] | 754.9 | [9.1] | 1008.0 | [31.9] | 678.9 | [30.0] | 842.8 | [13.9] |
| **SP** | | | | | | | | | | | | | |
| Max. vel | [°/s] | 10.2 | [0.0] | 110.2 | [0.5] | 110.8 | [0.8] | 110.7 | [0.7] | 70.8 | [0.5] | 150.7 | [0.6] |
| Max. acc | [°/s^2^] | 25.5 | [5.3] | 500.6 | [14.2] | 745.8 | [17.8] | 1037.6 | [43.5] | 679.9 | [27.9] | 846.1 | [20.6] |

*A slow profile was used to determine the ankle range of motion of participants. With Max. maximum, Med. Medium, vel velocity, acc acceleration*

**Table 2: Effects on the amount of muscle activation for typically developing (TD) children and children with spastic paresis (SP).** **Median and interquartile range values per group as well as statistical outcomes.**

|  | | | **ACCELERATION** | | | | | | | | | | | | | | | | | | | | | | **VELOCITY** | | | | | | | | | | | | | | | | | | | | | | | | | | | | | | |  |
| --- | --- | --- | --- | --- | --- | --- | --- | --- | --- | --- | --- | --- | --- | --- | --- | --- | --- | --- | --- | --- | --- | --- | --- | --- | --- | --- | --- | --- | --- | --- | --- | --- | --- | --- | --- | --- | --- | --- | --- | --- | --- | --- | --- | --- | --- | --- | --- | --- | --- | --- | --- | --- | --- | --- | --- | --- |
|  | | | **low** | | | | | **med** | | | | | | | **high** | | | | | |  | | | | **low** | | | | | | | | **med** | | | | | | | | | **high** | | | | | | |  | | | | | | | TD vs SP |
| Parameter | Unit | |  | |  | | |  | |  | | | | |  | | |  | | | p-val | | | |  | | | |  | | | |  | | | |  | | | | |  | | |  | | | | p-val | | | | | | |  |
| **TD** | | | | | | | | | | | | | | | | | | | | | | | | | | | | | | | | | | | | | | | | | | | | | | | | | | | | | | | | |
| Average EMG |  | |  | | |  | |  | | |  | | | | | |  |  | | | |  | |  | | |  | | | | |  | | | |  | |  | | | |  | | | |  |  | | | | | |  | | |  |
| GM | [uV] | | 1.58 | | | [2.33] | | 1.28 | | | | | [2.92] | | | | 1.49 | | [0.81] | | | 0.417 | |  | | | 1.09 | | | | [0.29] | | | | 1.28 | | [2.92] | | | | | 1.20 | | | [3.00] | | 0.197 | | | | | |  | | | **<0.001** |
| GL | [uV] | | 1.32 | | | [0.45] | | 1.25 | | | | | [0.59] | | | | 1.31 | | [0.16] | | | 0.883 | |  | | | 1.13 | | | | [0.64] | | | | 1.25 | | [0.59] | | | | | 1.23 | | | [0.26] | | 0.223 | | | | | |  | | | **<0.001** |
| SO | [uV] | | 1.31 | | | [1.93] | | 1.67 | | | | | [2.51] | | | | 1.31 | | [0.47] | | | 0.417 | |  | | | 1.17 | | | | [0.35] | | | | 1.67 | | [2.51] | | | | | 1.28 | | | [0.59] | | 0.417 | | | | | |  | | | **<0.001** |
| Peak EMG | | | | | | | | | | | | | | | | | | | | | | | | | | | | | | | | | | | | | | | | | | | | | | | | | | | | | | | | |
| GM | [uV] | | 4.36 | | | [12.00] | | 2.30 | | | | | [16.68] | | | | 3.44 | | [4.97] | | | 0.607 | |  | | | 1.89 | | | | [0.88] | | | | 2.30 | | [16.68] | | | | | 2.22 | | [18.81] | | | 0.607 | | | | |  | | | **<0.001** | |
| GL | [uV] | | 2.96 | | | [2.45] | | 2.25 | | | | | [2.29] | | | | 2.36 | | [0.73] | | | 0.883 | |  | | | 1.90 | | | | [2.16] | | | | 2.25 | | [2.29] | | | | | 2.13 | | [0.79] | | | 0.197 | | | | |  | | | **<0.001** | |
| SO | [uV] | | 2.50 | | | [10.33] | | 4.61 | | | | | [9.74] | | | | 2.40 | | [1.54] | | | 0.197 | |  | | | 1.92 | | | | [2.05] | | | | 4.61 | | [9.74] | | | | | 2.47 | | [1.89] | | | 0.417 | | | | |  | | | **<0.001** | |
| **SP** | | | | | | | | | | | | | | | | | | | | | | | | | | | | | | | | | | | | | | | | | | | | | | | | | | | | | | | | |
| Average EMG | | | | | | | | | | | | | | | | | | | | | | | | | | | | | | | | | | | | | | | | | | | | | | | | | | | | | | | | |
| GM | [uV] | | 5.28 | | [7.88] | | | 5.04 | | | | [5.68] | | | | 4.72 | | [4.13] | | | 0.368 | |  | | | 2.92 | | | | [4.56] | | | | 5.04 | | | [5.68] | | | | 6.07 | | [4.67] | | | | | **0.023** | | | lm | | |  | | |
| GL | [uV] | | 3.81 | | [3.71] | | | 3.10 | | | | [4.04] | | | | 3.27 | | [2.24] | | | **0.023** | |  | | | 2.62 | | | | [1.40] | | | | 3.10 | | | [4.04] | | | | 4.02 | | [3.64] | | | | | **0.013** | | | lh | | |  | | |
| SO | [uV] | | 5.07 | | [5.72] | | | 4.34 | | | | [5.08] | | | | 2.91 | | [4.44] | | | **0.023** | | lh | | | 3.02 | | | | [3.85] | | | | 4.34 | | | [5.08] | | | | 4.76 | | [5.50] | | | | | **0.025** | | | lm | | |  | | |
| Peak EMG | | | | | | | | | | | | | | | | | | | | | | | | | | | | | | | | | | | | | | | | | | | | | | | | | | | | | | | | |
| GM | | [uV] | | 19.88 | | | [21.90] | | 11.51 | | | | | [13.65] | | 11.96 | | | | [10.50] | | 0.116 |  | | | | | 8.41 | | [10.74] | | | | | 11.51 | | | | [13.65] | 16.81 | | | | | | [14.68] | | | | **0.023** | lh | | |  | | |
| GL | | [uV] | | 12.33 | | | [13.36] | | 9.45 | | | | | [14.18] | | 8.19 | | | | [7.47] | | **0.018** | lh | | | | | 6.41 | | [3.68] | | | | | 9.45 | | | | [14.18] | 15.68 | | | | | | [13.83] | | | | **0.006** | lh | | |  | | |
| SO | | [uV] | | 18.87 | | | [20.35] | | 16.64 | | | | | [16.62] | | 9.86 | | | | [18.82] | | **0.008** | lm, lh | | | | | 8.42 | | [16.03] | | | | | 16.64 | | | | [16.62] | 17.91 | | | | | | [12.16] | | | | **0.001** | lm,lh | | |  | | |

*Post hoc results are indicated when p<0.05 (with Tukey-Kramer correction for multiple comparison), with lm representing a significant difference between the low and medium conditions and lh a difference between the low and high conditions (no differences were found for mh). Results for testing between the grouped data of TD and SP are also given. With GM Gastrocnemius Medialis, GL Gastrocnemius Lateralis, SO soleus muscles.*

**Table 3: Percentage of detected EMG onsets in children with spastic paresis.**

|  | **ACC_LOW** | **A/V_MED** | **ACC_HIGH** | **VEL_LOW** | **VEL_HIGH** |
| --- | --- | --- | --- | --- | --- |
| **GM** | 81% | 85% | 77% | 73% | 77% |
| **GL** | 88% | 88% | 81% | 77% | 92% |
| **SO** | 85% | 77% | 81% | 77% | 81% |

*Percentage of onsets taken over all trials in the SP data per profile. With GM gastrocnemius medialis, GL gastrocnemius lateralis, SO soleus muscle.*

**Table 4: Effects on EMG burst characteristics in children with spastic paresis.** **Median and interquartile range values for group effects on acceleration and velocity as well as statistical outcomes.**

|  |  | **ACCELERATION** | | | | | | | | | **VELOCITY** | | | | | | | | | | | |
| --- | --- | --- | --- | --- | --- | --- | --- | --- | --- | --- | --- | --- | --- | --- | --- | --- | --- | --- | --- | --- | --- | --- |
|  |  | **low** | | **med** | | **high** | |  | | | **low** | | | **med** | | **high** | | |  | |  | |
| **Parameter** | **Unit** |  |  |  |  |  |  | | **p-val** |  |  |  |  | |  | |  |  | |  | |  |
| Total time from start stretch to EMG burst onset | | | | | | | | | | | | | | | | | | | | | | |
| GM | s | 0.27 | [0.11] | 0.13 | [0.06] | 0.11 | [0.04] | | **<0.001** | lm,lh,mh | 0.09 | [0.02] | 0.13 | | [0.06] | | 0.22 | [0.03] | | **<0.001** | | lm,lh,mh |
| GL | s | 0.30 | [0.11] | 0.17 | [0.06] | 0.13 | [0.04] | | **<0.001** | lm,lh,mh | 0.11 | [0.02] | 0.17 | | [0.05] | | 0.25 | [0.04] | | 0.070 | | lm |
| SO | s | 0.26 | [0.13] | 0.17 | [0.07] | 0.13 | [0.05] | | **<0.001** | lm,lh,mh | 0.10 | [0.02] | 0.17 | | [0.07] | | 0.22 | [0.06] | | **0.006** | | lm,lh,mh |
| Peak EMG burst | | | | | | | | | | | | | | | | | | | | | | |
| GM | % | 36.0 | [24.7] | 38.0 | [22.4] | 46.2 | [16.9] | | 0.670 |  | 32.4 | [30.0] | 38.0 | | [22.4] | | 55.3 | [25.4] | | 0.156 | |  |
| GL | % | 33.3 | [12.5] | 37.4 | [11.2] | 37.6 | [15.1] | | 0.497 |  | 23.5 | [12.5] | 37.4 | | [11.2] | | 36.7 | [12.1] | | **0.042** | | lm |
| SO | % | 41.3 | [33.8] | 31.1 | [19.6] | 39.6 | [21.0] | | 0.741 |  | 34.1 | [11.2] | 31.1 | | [19.6] | | 32.6 | [27.6] | | 0.311 | |  |

*Post hoc results are indicated when p<0.05 (with Tukey-Kramer correction for multiple comparison), with lm representing a significant difference between the low and medium conditions, lh a difference between the low and high conditions and mh between medium and high. With max. GM Gastrocnemius Medialis, GL Gastrocnemius Lateralis, SO soleus muscles.*
